# A cross-sectional study assessing the relationship between non-alcoholic fatty liver disease and periodontal disease

**DOI:** 10.1038/s41598-022-17917-2

**Published:** 2022-08-10

**Authors:** Satsuki Sato, Yohei Kamata, Takaomi Kessoku, Tomoko Shimizu, Takashi Kobayashi, Takeo Kurihashi, Shogo Takashiba, Kazu Hatanaka, Nobushiro Hamada, Toshiro Kodama, Takuma Higurashi, Masataka Taguri, Masato Yoneda, Haruki Usuda, Koichiro Wada, Atsushi Nakajima, Toshiya Morozumi, Masato Minabe

**Affiliations:** 1 Department of Highly Advanced Oral Stomatology, Yokohama Clinic, Kanagawa Dental University, 3-31-6 Tsuruya-cho, Kanagawa, Yokohama, Kanagawa 221-0835 Japan; 2 Department of Gastroenterology and Hepatology, Yokohama City University Graduate School of Medicine, 3-9 Fukuura, Kanazawa-ku, Yokohama, 236-0004 Japan; 3 Department of Internal Medicine, Yokohama Clinic, Kanagawa Dental University, 3-31-6 Tsuruya-cho, Kanagawa, Yokohama, Kanagawa 221-0835 Japan; 4 Department of Pathophysiology-Periodontal Science, Okayama University Graduate School of Medicine, Dentistry and Pharmaceutical Sciences, 2-5-1 Shikata-cho, Kita-ku, Okayama, 700-8525 Japan; 5 Division of Microbiology, Department of Oral Science Graduate School of Dentistry, Kanagawa Dental University, 82 Inaoka-cho, Yokosuka, Kanagawa 238-8580 Japan; 6 Department of Implantology and Periodontology, Graduate School of Dentistry, Kanagawa Dental University, 3-31-6 Tsuruya-cho, Kanagawa, Yokohama, Kanagawa 221-0835 Japan; 7 Department of Biostatistics, Yokohama City University Graduate School of Medicine, 3-9 Fukuura, Kanazawa-ku, Yokohama, Kanagawa 236-0004 Japan; 8 Department of Pharmacology, Shimane University School of Medicine, 89-1 Enya-cho Izumo, Shimane, 693-0581 Japan; 9 Division of Periodontology, Department of Oral Interdisciplinary Medicine, Graduate School of Dentistry, Kanagawa Dental University, 82 Inaoka-cho, Yokosuka, Kanagawa 238-8580 Japan

**Keywords:** Dental diseases, Gastroenterology, Saliva

## Abstract

The risk factors for non-alcoholic fatty liver disease (NAFLD) progression are not completely known. *Porphyromonas* *gingivalis* infection is a risk factor for systemic diseases. We investigated the association of *P.* *gingivalis* infection with the risk of non-alcoholic steatohepatitis progression. Here, hematological tests, periodontal examination, and saliva collection were performed for 164 patients with NAFLD. *P.* *gingivalis* was identified in saliva using polymerase chain reaction. Hepatic steatosis and stiffness were evaluated using vibration-controlled transient elastography (VCTE) and magnetic resonance imaging. In patients with NAFLD, *P.* *gingivalis* positivity (*P.* *gingivalis* ratio ≥ 0.01%) in saliva correlated with liver stiffness determined using magnetic resonance elastography (MRE; p < 0.0001). A *P.* *gingivalis* ratio of 0.01% corresponds to 100,000 cells/mL and indicates the proportion of *P.* *gingivalis* in the total number of bacteria in the oral cavity. Patients with NAFLD and advanced fibrosis on MRE showed significantly elevated endotoxin activity; those who had > 10 periodontal pockets with depths ≥ 4 mm had significantly increased hepatic stiffness on both VCTE and MRE.

## Introduction

Non-alcoholic fatty liver disease (NAFLD) is now recognized as one of the most common chronic liver diseases^1^. It is common knowledge that people with high alcohol consumption rates develop fatty livers; however, recent studies have found that fatty liver also occurs in individuals who consume little or no alcohol^2^. NAFLD is an umbrella term for simple fatty liver (in which fat is deposited in the liver with little or no inflammation) and non-alcoholic steatohepatitis (NASH), which is a risk factor for fibrosis and liver cancer. It is believed that insulin resistance, oxidative stress, inflammatory cytokines, and other factors contribute to the progression of simple fatty liver to NASH, which finally leads to cirrhosis and hepatocellular carcinoma. Thus, NAFLD is an important disease, affecting approximately 30% of the population in Japan^3,4^. It has been reported that NAFLD is closely associated with the constituents of metabolic syndrome, such as obesity^5^, type 2 diabetes mellitus^6^, hypertension^7^, and hyperlipidemia^8^. In addition, this disease has been noted to co-occur with other diseases such as sleep apnea syndrome^9^ and hypothyroidism^10^. It is clear that diet modification is effective in patients with NAFLD^11–15^; however, no gold standard has been established for drug therapy^16–21^.

Periodontal disease refers to chronic inflammation of the supporting tissues of teeth. It is associated with many metabolic diseases such as diabetes mellitus^22^, cardiovascular disease^23^, and NAFLD^24^. In animal studies, periodontal disease has been found to induce increased blood concentrations of proinflammatory molecules and increase the oxidative stress that occurs because of these mediators^25–34^. These studies support the hypothesis that periodontal disease may be a risk factor for NAFLD. Animal studies have demonstrated a relationship between *Porphyromonas* *gingivalis* and fatty liver disease^35^, and human studies have shown the involvement of *P.* *gingivalis* in NAFLD progression^36^; however, the relationship between NAFLD and periodontal disease in humans remains unclear.

We hypothesized that deep periodontal pockets and *P.* *gingivalis* bacterial content would be associated with hepatic fibrogenesis. Therefore, this study aimed to investigate the relationship between the pathogenesis of NAFLD and intraoral findings.

## Results

According to the sample size calculation, 164 patients who met the eligibility criteria were identified and included between August 2015 and April 2019.

Of the 164 participants, *P.* *gingivalis* represented ≥ 0.01% and < 0.01% of all bacteria in the saliva of 45 and 119 patients, respectively.

A *P.* *gingivalis* ratio of 0.01% corresponds to approximately 100,000 cells/mL and indicates the ratio of *P.* *gingivalis* to the total number of bacteria in the oral cavity quantified by performing polymerase chain reaction (PCR) of the DNA obtained from saliva samples^37,38^. The baseline demographics and disease characteristics of the patient groups with a *P.* *gingivalis* ratio ≥ 0.01% and a *P.* *gingivalis* *ratio* < 0.01% in saliva were almost similar (Table 1). Liver stiffness measured by magnetic resonance elastography (MRE) was significantly higher in the group with a *P.* *gingivalis* ratio ≥ 0.01% than in the group with a *P.* *gingivalis* ratio < 0.01% in saliva (3.0 ± 1.3 vs. 3.7 ± 1.0, p = 0.0064, Table 1). In addition, there was a significant difference between these groups in terms of the level of the inflammatory marker, ferritin (215 ± 198 vs. 282 ± 142, p = 0.04, Table 1).

**Table 1:** Baseline characteristics of the trial population.

Variables	All (n = 164)	*P.* *gingivalis* < 0.01% (n = 119)	*P.* *gingivalis* ≥ 0.01% (n = 45)	p-value
**Demographics**				
Age (years)	57 (15)	55 (15)	60 (15)	0.043
Male (%)	92 (56)	70 (59)	22 (49)	0.255
**Comorbidities**				
Type 2 diabetes (%)	56 (34)	41 (34)	15 (33)	0.893
Hyperlipidemia (%)	59 (36)	46 (39)	13 (29)	0.248
Hypertension (%)	57 (35)	43 (36)	14 (31)	0.55
Hyperuricemia (%)	15 (9)	9 (8)	6 (13)	0.255
Cardiovascular disease (%)	6 (4)	6 (5)	0 (0)	0.126
Thyroid disease (hypothyroidism) (%)	3 (2)	2 (2)	1 (2)	0.819
**Concomitant drug use**				
Anti-diabetic (%)	57 (35)	41 (34)	16 (35)	0.896
DPP4-inhibitor (%)	39 (24)	26 (22)	13 (29)	0.348
Metformin (%)	31 (19)	21 (18)	10 (22)	0.507
Sulfonylurea (%)	17 (10)	11 (9)	6 (13)	0.446
Anti-lipidemic (%)	56 (34)	43 (36)	13 (29)	0.386
Anti-hypertensive (%)	56 (34)	42 (35)	14 (31)	0,617
Anti-platelet (%)	0 (0)	0 (0)	0 (0)	
Laxatives, regular use (%)	0 (0)	0 (0)	0 (0)	
**Liver imaging**				
CAP on VCTE (dB/m)	305 (60)	310 (56)	295 (69)	0.168
LSM on VCTE (kPa)	7.7 (4.3)	7.3 (4.4)	8.6 (3.8)	0.114
Liver fat content on MRI-PDFF (%)	12.4 (6.6)	13.0 (6.6)	10.8 (6.4)	0.091
Liver stiffness on MRE (kPa)	3.2 (1.2)	3.0 (1.3)	3.7 (1.0)	0.0064
**Endotoxin**				
EAA (× 10^2^)	0.1 (0.09)	0.1 (0.08)	0.2 (0.08)	0.001
**Metabolic factors**				
Weight (kg)	72.6 (15.6)	73 (16)	71 (15)	0.473
BMI (kg/m^2^)	27 (5.3)	27.3 (5.0)	26.1 (6.0)	0.177
Glucose (mg/dL)	109 (35)	112 (38)	101 (26)	0.093
Insulin (μU/mL)	20 (28)	21 (31)	15 (18)	0.183
HOMA-R	6 (10)	7 (11)	4 (8)	0.2
**Liver function**				
Platelet count	22 (8)	22 (9)	20 (4)	0.182
AST (U/L)	37 (21)	35 (21)	42 (22)	0.056
ALT (U/L)	50 (38)	48 (40)	54 (30)	0.434
GGT (U/L)	77 (97)	73 (69)	86 (147)	0.447
ALP (U/L)	241 (91)	238 (88)	250 (99)	0.476
T.Bil (mg/dL)	1.2 (4.8)	1.3 (5.7)	0.7 (0.3)	0.428
**Lipids**				
Tcho (mg/dL)	198 (40)	199 (38)	196 (44)	0.591
LDL-C (mg/dL)	119 (85)	121 (98)	112 (35)	0.56
HDL-C (mg/dL)	43 (82)	40 (95)	50 (15)	0.459
TG (mg/dL)	182 (130)	189 (143)	162 (86)	0.244
**Inflammatory markers**				
hsCRP (mg/L)	0.2 (0.3)	0.2 (0.4)	0.1 (0.2)	0.512
Ferritin (ng/mL)	233 (187)	215 (198)	282 (142)	0.042
**Fibrosis marker**				
Type IV collagen 7s (ng/mL)	4.2 (1.0)	4.2 (1.0)	4.3 (1.0)	0.483
**Periodontal assessment**				
PPD (mm)	2.5 (0.5)	2.4 (0.5)	2.7 (0.5)	0.0091
CAL (mm)	2.5 (0.5)	2.4 (0.5)	2.7 (0.5)	0.0091
BOP (site)	23 (30)	19 (28)	32 (31)	0.0118
Stability of teeth	0	0	0	
PPD ≥ 4 mm (site)	14 (23)	12 (22)	22 (23)	0.01
Oral bacteria, *P.* *gingivalis* (× 10^6^ cells/mL)	0.4 (1.0)	0.2 (0.5)	1.2 (1.6)	< 0.0001
Antibody titer for *P.* *gingivalis* FDC381	0.6 (1.8)	0.5 (1.9)	0.8 (1.4)	0.28
Antibody titer for *P.* *gingivalis* SU63	0.9 (2.0)	0.5 (1.5)	2.0 (2.6)	< 0.0001
IMT mean (R)	0.9 (0.3)	0.9 (0.3)	1.0 (0.3)	0.0402
IMT mean (L)	0.9 (0.3)	0.9 (0.2)	1.0 (0.3)	0.008
IMT max (R)	1.1 (0.5)	1.1 (0.5)	1.2 (0.4)	0.239
IMT max (L)	1.1 (0.5)	1.1 (0.6)	1.2 (0.5)	0.346

*ALP* alkaline phosphatase, *ALT* alanine aminotransferase, *AST* aspartate aminotransferase, *BMI* body mass index, *BOP* bleeding on probing, *CAL* clinical attachment level, *CAP* controlled attenuation parameter, *DPP4* dipeptidyl peptidase-4, *EAA* endotoxin activity assay, *GGT* gamma-glutamyl transferase, *HDL-C* high-density lipoprotein cholesterol, *HOMA-R* homeostasis model assessment-estimated insulin resistance, *hsCRP* high-sensitivity C-reactive protein, *IMT* *(R:* *right,* *L:* *left)* intima-media thickness (R, L), *LDL-C* low-density lipoprotein cholesterol, *LSM* liver stiffness measurement, *MRE* magnetic resonance elastography, *MRI* magnetic resonance imaging, *PDFF* proton density fat fraction, *PPD* periodontal pocket depth, *T.Bil* total bilirubin, *Tcho* total cholesterol, *TG* triglycerides, *VCTE* vibration-controlled transient elastography.

^a^Data are reported as means (SDs) or numbers (percentages). The p-values were determined using the t-test.

**Figure 1:**
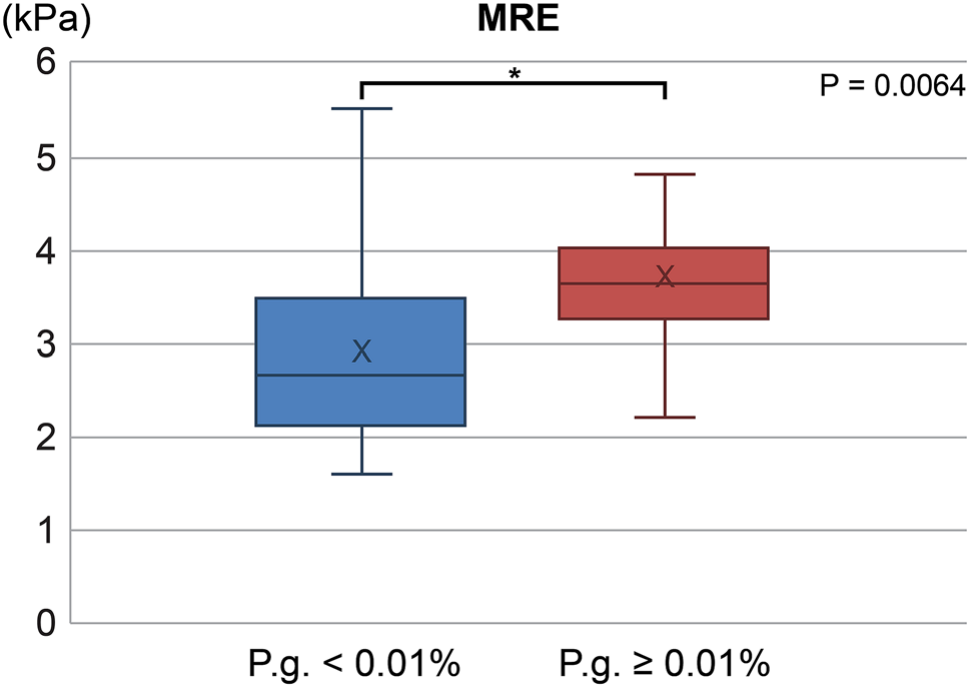
Liver stiffness in groups of patients with < 0.01% and ≥ 0.01% *P.* *gingivalis* in saliva. MRE, magnetic resonance elastography.

The baseline periodontal pocket depth (PPD) [*P.* *gingivalis* in saliva ≥ 0.01% group, mean (standard deviation, SD): 2.7 (0.5) mm; *P.* *gingivalis* in saliva < 0.01% group, 2.4 (0.5) mm], PPD ≥ 4 mm [%; *P.* *gingivalis* in saliva ≥ 0.01% group, 43 (96%) patients; *P.* *gingivalis* in saliva < 0.01% group, 119 (73%) patients], and antibody titer for *P.* *gingivalis* SU63 [*P.* *gingivalis* in saliva ≥ 0.01% group mean (SD): 2.0 (2.6); *P.* *gingivalis* in saliva < 0.01% group: 0.5 (1.5)] indicated that this study population had a stable periodontal disease condition (Table 1, Fig. 1).

**Figure 2:**
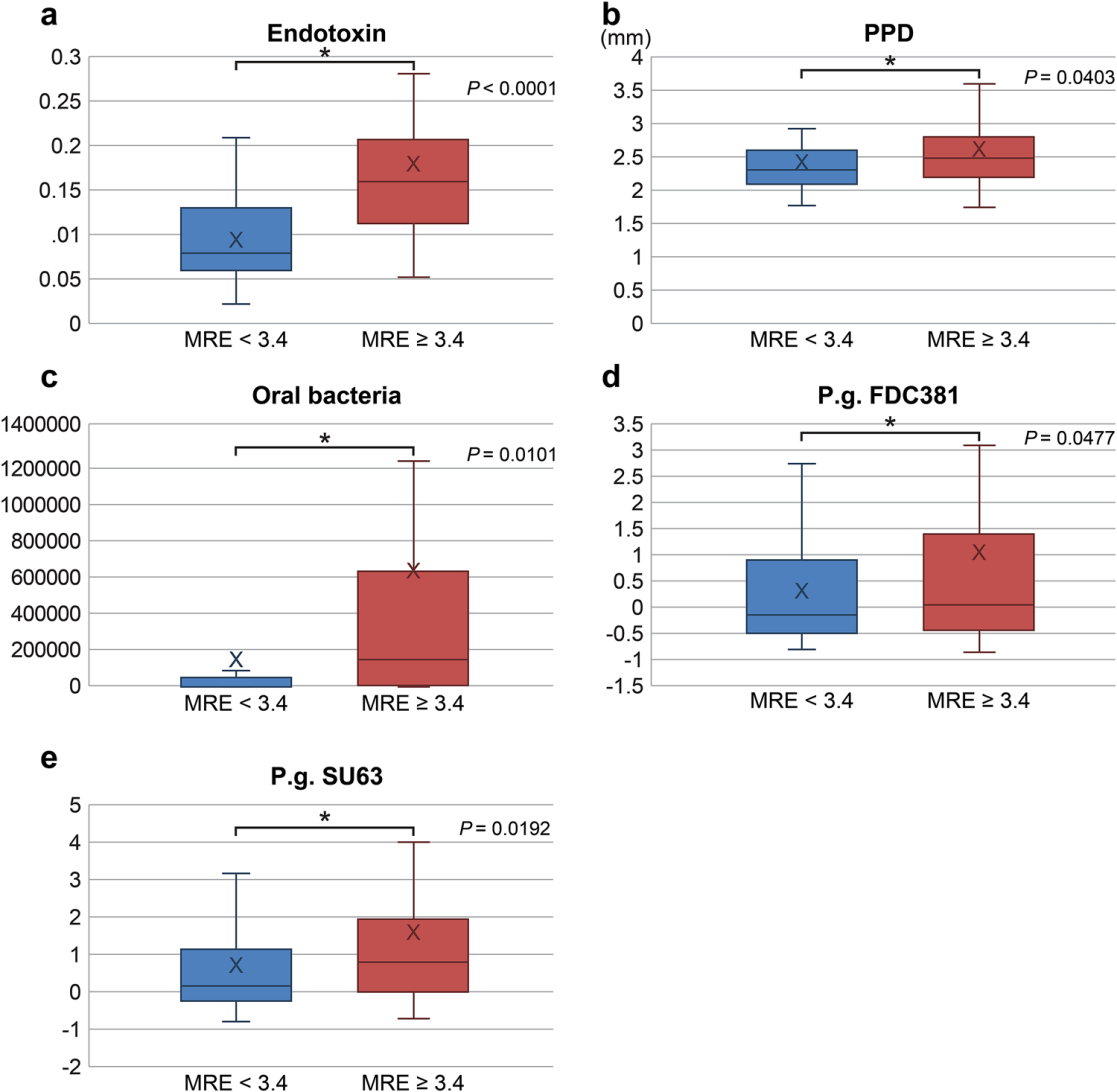
Endotoxin activity (**a**), PPD (**b**), periodontal disease-causing bacteria, *P.* *gingivalis* (**c**), antibody titer for *P.* *gingivalis* FDC381 (**d**), and antibody titer for *P.* *gingivalis* SU63 (**e**) in the groups with MRE < 3.4 kPa and MRE ≥ 3.4 kPa. *MRE* magnetic resonance elastography, *PPD* periodontal pocket depth.

The high elasticity group (MRE ≥ 3.4 kPa) included 64 participants, and the low elasticity group (MRE < 3.4 kPa) included 100 participants. We found significant differences between these two groups in terms of the presence of oral *P.* *gingivalis* (6.0 ± 1.3 vs. 0.2 ± 0.5 × 10^6^ cells/mL, respectively; p < 0.01), results of endotoxin activity assay (0.2 ± 0.12 vs. 0.1 ± 0.05, respectively; p < 0.0001), and antibody titers for *P.* *gingivalis* FDC381 (1.0 ± 2.4 vs. 0.3 ± 1.1, respectively; p < 0.0477) and SU63 (1.6 ± 2.6 vs. 0.7 ± 1.7, respectively; p < 0.019). These data suggest that an increased liver stiffness (determined by MRE) is associated with a high number of *P.* *gingivalis* bacteria in the oral cavity, increased endotoxin levels, and high antibody titers for *P.* *gingivalis* FDC381 and SU63 (Table 2, Fig. 2a–e).

**Table 2:** Baseline characteristics of the trial population.

Variables	All (n = 164)	MRE < 3.4 kPa (n = 100)	MRE ≥ 3.4 kPa (n = 64)	p-value
**Demographics**				
Age (years)	57 (15)	56 (14)	60 (14)	
Male (%)	92 (56)	57 (57)	25 (39)	
**Comorbidities**				
Type 2 diabetes (%)	56 (34)	33 (33)	23 (36)	
Hyperlipidemia (%)	59 (36)	38 (38)	21 (31)	
Hypertension (%)	57 (35)	35 (35)	22 (34)	
Hyperuricemia (%)	15 (9)	9 (9)	6 (9)	
Cardiovascular disease (%)	6 (4)	3 (3)	3 (0)	
Thyroid disease (hypothyroidism) (%)	3 (2)	1 (1)	2 (0)	
**Concomitant drug use**				
Anti-diabetic (%)	57 (35)	32 (32)	25 (35)	
DPP4-inhibitor (%)	39 (24)	22 (22)	17 (27)	
Metformin (%)	31 (19)	21 (21)	10 (16)	
Sulfonylurea (%)	17 (10)	10 (10)	7 (11)	
Anti-lipidemic (%)	56 (34)	37 (37)	19 (30)	
Anti-hypertensive (%)	56 (34)	34 (34)	22 (34)	
Anti-platelet (%)	0 (0)	0 (0)	0 (0)	
Laxatives, regular use (%)	0 (0)	0 (0)	0 (0)	
**Liver imaging**				
CAP on VCTE (dB/m)	305 (60)	309 (57)	297 (67)	
LSM on VCTE (kPa)	7.7 (4.3)	6.3 (3.2)	10.9 (4.8)	< 0.0001
Liver fat content on MRI-PDFF (%)	12.4 (6.6)	12.5 (7.2)	12.2 (5.5)	
Liver stiffness on MRE (kPa)	3.2 (1.2)	2.4 (0.5)	4.3 (1.3)	< 0.0001
**Endotoxin**				
EAA (× 10^2^)	0.1 (0.09)	0.1 (0.05)	0.2 (0.12)	< 0.0001
**Metabolic factors**				
Weight (kg)	72.6 (15.6)	75 (15)	69 (15)	0.022
BMI (kg/m^2^)	27 (5.3)	27.9 (4.9)	26.6 (4.5)	
Glucose (mg/dL)	109 (35)	113 (33)	111 (44)	
Insulin (μU/mL)	20 (28)	20 (26)	18 (18)	
HOMA-R	6 (10)	6 (8)	6 (11)	
**Liver function**				
Platelet count	22 (8)	23 (9)	19 (6)	0.0239
AST (U/L)	37 (21)	36 (17)	46 (24)	0.0065
ALT (U/L)	50 (38)	54 (44)	55 (27)	
GGT (U/L)	77 (97)	71 (70)	100 (145)	
ALP (U/L)	241 (91)	236 (58)	268 (135)	
T.Bil (mg/dL)	1.2 (4.8)	1.5 (7.0)	0.8 (0.6)	
**Lipids**				
Tcho (mg/dL)	198 (40)	199 (39)	202 (40)	
LDL-C (mg/dL)	119 (85)	127 (119)	115 (29)	
HDL-C (mg/dL)	43 (82)	34 (117)	51 (20)	
TG (mg/dL)	182 (130)	192 (140)	175 (99)	
**Inflammatory markers**				
hsCRP (mg/L)	0.2 (0.3)	0.2 (0.4)	0.2 (0.3)	
Ferritin (ng/mL)	233 (187)	223 (207)	271 (147)	
**Fibrosis marker**				
Type IV collagen 7s (ng/mL)	4.2 (1.0)	4.0 (0.7)	4.9 (1.2)	< 0.0001
**Periodontal assessment**				
PPD (mm)	2.5 (0.5)	2.4 (0.5)	2.6 (0.6)	0.0403
CAL (mm)	2.5 (0.5)	2.4 (0.5)	2.6 (0.6)	0.0403
BOP (site)	23 (30)	13 (18)	38 (40)	< 0.0001
Stability of teeth	0	0	0	
PPD ≥ 4 mm (site)	10 (14)	6 (12)	16 (17)	0.0004
Oral bacteria, *P.* *gingivalis* (× 10^6^ cells/mL)	0.4 (1.0)	0.2 (0.5)	6.0 (1.3)	0.0101
Antibody titer for *P.* *gingivalis* FDC381	0.6 (1.8)	0.3 (1.1)	1.0 (2.4)	0.0477
Antibody titer for *P.* *gingivalis* SU63	0.9 (2.0)	0.7 (1.7)	1.6 (2.6)	0.0192
IMT mean (R)	0.9 (0.3)	0.8 (0.2)	1.1 (0.3)	< 0.0001
IMT mean (L)	0.9 (0.3)	0.8 (0.2)	1.1 (0.3)	< 0.0001
IMT max (R)	1.1 (0.5)	1.0 (0.4)	1.4 (0.5)	< 0.0001
IMT max (L)	1.1 (0.5)	1.0 (0.5)	1.5 (0.6)	< 0.0001

*ALP* alkaline phosphatase, *ALT* alanine aminotransferase, *AST* aspartate aminotransferase, *BMI* body mass index, *BOP* bleeding on probing, *CAP* controlled attenuation parameter, *CAL* clinical attachment level, *DPP4* dipeptidyl peptidase-4, *EAA* endotoxin activity assay, *GGT* gamma-glutamyl transferase, *HDL-C* high-density lipoprotein cholesterol, *HOMA-R* homeostasis model assessment-estimated insulin resistance, *hsCRP* high-sensitivity C-reactive protein, *IMT* *(R:* *right,* *L:* *left)* intima-media thickness (R, L), *LDL-C* low-density lipoprotein cholesterol, *LSM* liver stiffness measurement, *MRE* magnetic resonance elastography, *MRI* magnetic resonance imaging, *PDFF* proton density fat fraction, *PPD* periodontal pocket depth, *T.Bil* total bilirubin, *Tcho* total cholesterol, *TG* triglycerides, *VCTE* vibration-controlled transient elastography.

^a^Data are reported as means (SDs) or numbers (percentages). P-values were determined using the t-test.

**Figure 3:**
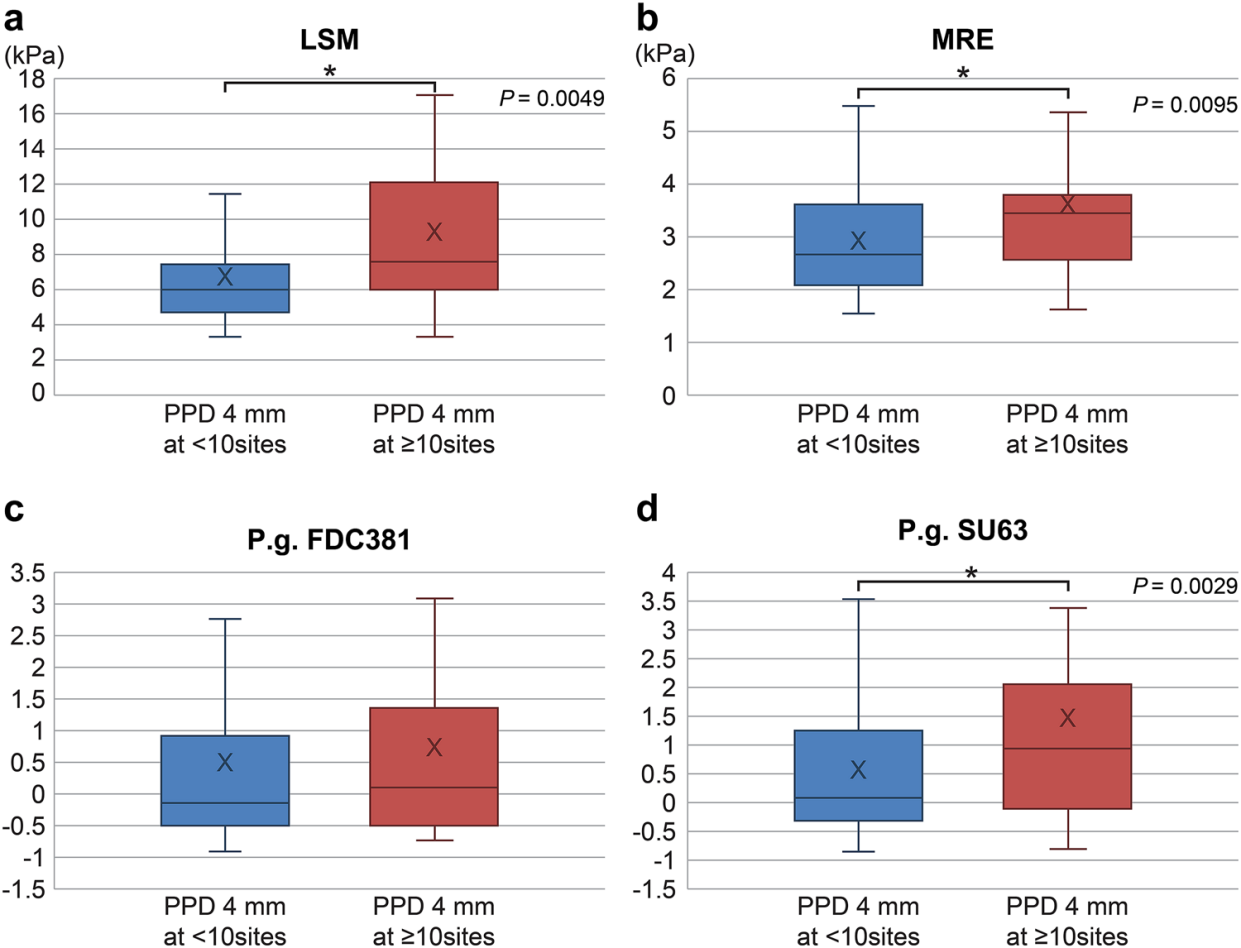
LSM (**a**), MRE (**b**), antibody titer for *P.* *gingivalis* FDC381 (**c**), and antibody titer for *P.* *gingivalis* SU63 (**d**) in patients with periodontal pockets ≥ 4 mm at a depth of < 10 mm or ≥ 10 sites. *LSM* liver stiffness measurement, *MRE* magnetic resonance elastography, *PPD* periodontal pocket depth.

Among the 164 study participants, 52 and 112 had less than 10 (3 ± 3 sites) and more than 10 (26 ± 17 sites) periodontal pockets with depths ≥ 4 mm, respectively. The liver stiffness values were significantly higher in the group with > 10 PPD sites than in the corresponding group with < 10 PPD sites (3.6 ± 1.5 vs. 3.0 ± 1.0 kPa, respectively; p < 0.0095; Table 3, Fig. 3a–d). The mean of PPD ≥ 4 mm was 4.0 mm (± 0.8). Moreover, there were significant differences between these groups regarding the periodontal conditions. These data indicate that liver stiffness increases in case of a greater number of deep periodontal pockets.

**Table 3:** Baseline characteristics of the study population.

Variables	All (n = 164)	PPD ≥ 4 mm at < 10 sites (n = 112)	PPD ≥ 4 mm at ≥ 10 sites (n = 52)	p-value
**Demographics**				
Age (years)	57 (15)	57 (15)	56 (13)	
Male (%)	92 (56)	66 (59)	26 (50)	
**Comorbidities**				
Type 2 diabetes (%)	56 (34)	36 (32)	20 (38)	
Hyperlipidemia (%)	59 (36)	42 (38)	17 (33)	
Hypertension (%)	57 (35)	40 (36)	17 (33)	
Hyperuricemia (%)	15 (9)	10 (9)	5 (10)	
Cardiovascular disease (%)	6 (4)	5 (4)	1 (2)	
Thyroid disease (hypothyroidism) (%)	3 (2)	1 (1)	2 (4)	
**Concomitant drug use**				
Anti-diabetic (%)	57 (35)	37 (33)	20 (38)	
DPP4-inhibitor (%)	39 (24)	24 (22)	15 (29)	
Metformin (%)	31 (19)	20 (18)	11 (21)	
Sulfonylurea (%)	17 (10)	10 (9)	7 (13)	
Anti-lipidemic (%)	56 (34)	40 (36)	16 (31)	
Anti-hypertensive (%)	56 (34)	40 (36)	16 (31)	
Anti-platelet (%)	0 (0)	0 (0)	0 (0)	
Laxatives, regular use (%)	0 (0)	0 (0)	0 (0)	
**Liver imaging**				
CAP on VCTE (dB/m)	305 (60)	305 (56)	304 (68)	
LSM on VCTE (kPa)	7.7 (4.3)	7.0 (3.9)	9.2 (4.8)	0.0049
Liver fat content on MRI-PDFF (%)	12.4 (6.6)	12.4 (6.8)	12.1 (6.3)	
Liver stiffness on MRE (kPa)	3.2 (1.2)	3.0 (1.0)	3.6 (1.5)	0.0095
**Endotoxin**				
EAA (× 10^2^)	0.1 (0.09)	0.1 (0.05)	0.2 (0.13)	< 0.0001
**Metabolic factors**				
Weight (kg)	72.6 (15.6)	72 (16)	72 (16)	
BMI (kg/m^2^)	27 (5.3)	27.0 (4.9)	26.7 (6.1)	
Glucose (mg/dL)	109 (35)	109 (32)	106 (40)	
Insulin (μU/mL)	20 (28)	21 (32)	17 (19)	
HOMA-R	6 (10)	6 (11)	6 (11)	
**Liver function**				
Platelet count	22 (8)	22 (9)	21 (6)	
AST (U/L)	37 (21)	38 (22)	35 (19)	
ALT (U/L)	50 (38)	51 (40)	45 (28)	
GGT (U/L)	77 (97)	80 (109)	65 (54)	
ALP (U/L)	241 (91)	256 (102)	231 (32)	
T.Bil (mg/dL)	1.2 (4.8)	1.3 (5.8)	0.8 (0.6)	
**Lipids**				
Tcho (mg/dL)	198 (40)	201 (39)	193 (43)	
LDL-C (mg/dL)	119 (85)	124 (101)	108 (31)	
HDL-C (mg/dL)	43 (82)	40 (98)	49 (19)	
TG (mg/dL)	182 (130)	185 (135)	178 (126)	
**Inflammatory markers**				
hsCRP (mg/L)	0.2 (0.3)	0.2 (0.4)	0.2 (0.3)	
Ferritin (ng/mL)	233 (187)	228 (200)	241 (151)	
**Fibrosis marker**				
Type IV collagen 7s (ng/mL)	4.2 (1.0)	4.2 (0.9)	4.4 (1.2)	
**Periodontal assessment**				
PPD (mm)	2.5 (0.5)	2.3 (0.3)	3.0 (0.5)	< 0.0001
CAL (mm)	2.5 (0.5)	2.3 (0.3)	3.0 (0.5)	< 0.0001
BOP (site)	23 (30)	12 (15)	47 (40)	< 0.0001
Stability of teeth	0	0	0	
PPD ≥ 4 mm (site)	10 (14)	3 (3)	26 (17)	< 0.0001
Oral bacteria, *P.* *gingivalis* (× 10^6^ cells/mL)	0.4 (1.0)	0.1 (0.3)	0.9 (1.6)	< 0.0001
Antibody titer for *P.* *gingivalis* FDC381	0.6 (1.8)	0.5 (1.5)	0.8 (2.4)	
Antibody titer for *P.* *gingivalis* SU63	0.9 (2.0)	0.6 (1.3)	1.5 (2.5)	0.0029
IMT mean (R)	0.9 (0.3)	0.8 (0.2)	1.2 (0.2)	< 0.0001
IMT mean (L)	0.9 (0.3)	0.8 (0.2)	1.1 (0.3)	< 0.0001
IMT max (R)	1.1 (0.5)	1.0 (0.4)	1.5 (0.4)	< 0.0001
IMT max (L)	1.1 (0.5)	1.0 (0.5)	1.5 (0.5)	< 0.0001

*ALP* alkaline phosphatase, *ALT* alanine aminotransferase, *AST* aspartate aminotransferase, *BMI* body mass index, *BOP* bleeding on probing, *CAP* controlled attenuation parameter, *CAL* clinical attachment level, *DPP4* dipeptidyl peptidase-4, *EAA* endotoxin activity assay, *GGT* gamma-glutamyl transferase, *HDL-C* high-density lipoprotein cholesterol, *HOMA-R* homeostasis model assessment-estimated insulin resistance, *hsCRP* high-sensitivity C-reactive protein, *IMT* *(R:* *right,* *L:* *left)* intima-media thickness (R, L), *LDL-C* low-density lipoprotein cholesterol, *LSM* liver stiffness measurement, *MRE* magnetic resonance elastography, *MRI* magnetic resonance imaging, *PDFF* proton density fat fraction, *PPD* periodontal pocket depth, *T.Bil* total bilirubin, *Tcho* total cholesterol, *TG* triglycerides, *VCTE* vibration-controlled transient elastography.

^a^Data are reported as means (SDs) or numbers (percentages). The p-values were determined using the t-test.

The results of logistic regression analysis using MRE ≥ 3.4 kPa as the dependent variable for all participants are shown in Table 4. The prevalence of NAFLD in advanced fibrosis was associated with weight [odds ratio (OR) = 0.948; p = 0.011], bleeding on probing (BOP) (OR = 1.045; p = 0.001), *P.* *gingivalis* ≥ 0.01 (OR = 4.05; p = 0.04), intima-media thickness (IMT) mean (OR = 1.21; p = 0.03), and PPD (OR = 2.045; p = 0.026).

**Table 4:** Factors associated with NAFLD in advanced fibrosis.

Covariate	Odds ratio	95% confidence interval	p-value
Endotoxin	1.050	0.946–1.166	0.337
Weight (kg)	0.948	0.907–0.991	0.011
Age	0.998	0.951–1.048	0.954
Platelet count (× 10^4^/μL)	0.977	0.900–1.060	0.523
AST (U/L)	1.013	0.984–1.044	0.341
Type VII collagen 7s (ng/mL)	1.705	0.889–3.271	0.098
BOP	1.045	1.013–1.079	0.001
*P.* *gingivalis* ≥ 0.01%	4.05	1.056–15.52	0.04
IMT mean	1.21	1.056–1.387	0.003
PPD (mm)	2.045	0.875–1.393	0.026

*AST* aspartate aminotransferase, *BOP* bleeding on probing, *IMT* intima-media thickness, *NAFLD* non-alcoholic fatty liver disease, *PPD* periodontal pocket depth.

**Figure 4:**
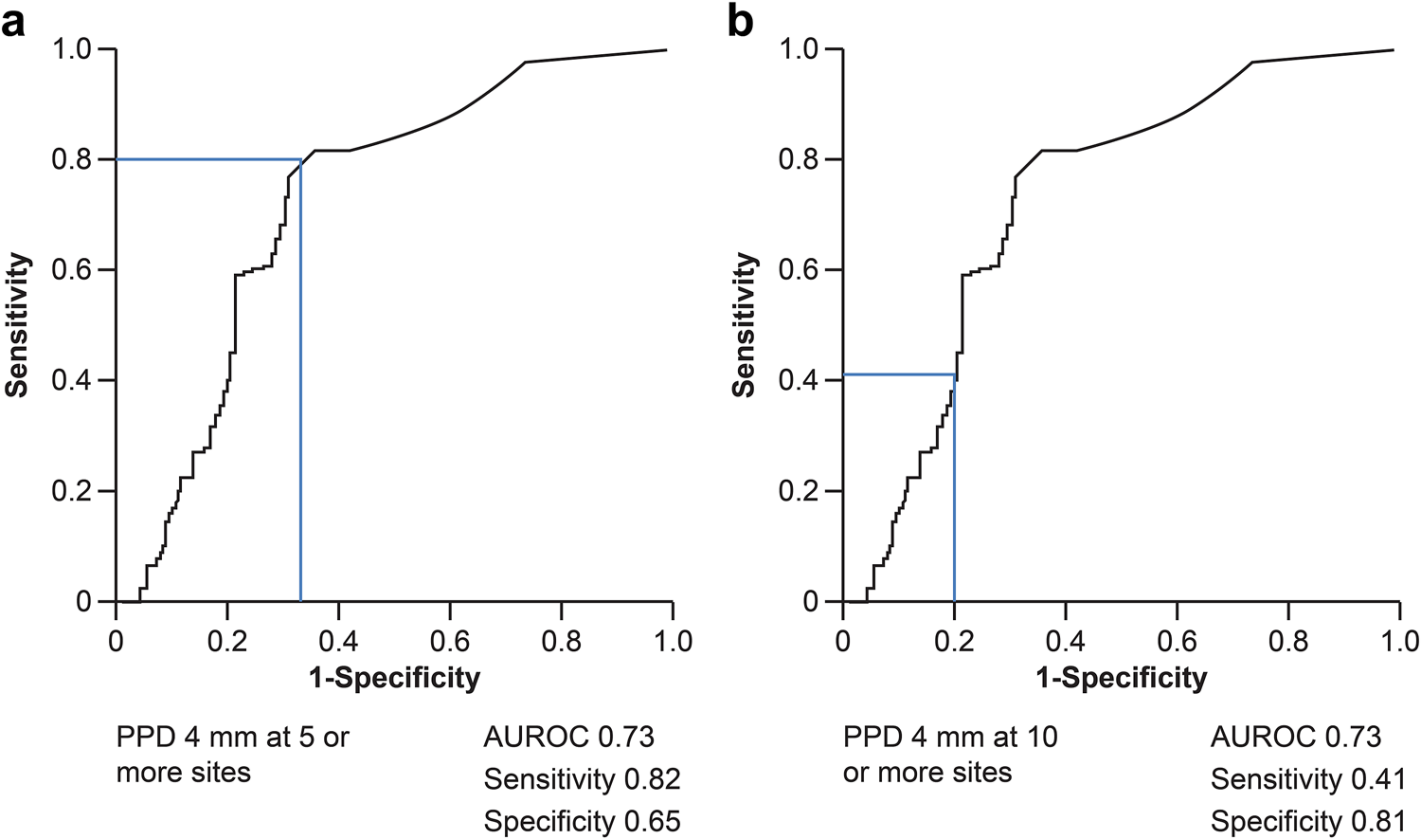
AUROC curves for patients with PPDs ≥ 4 mm at ≥ 5(**a**) and ≥ 10 (**b**) sites. *AUROC* area under the receiver operating characteristic, *PPD* periodontal pocket depth.

We also constructed receiver operating characteristic curves for patients using at least 0.01% *P.* *gingivalis* in saliva and the number of periodontal pockets with depths ≥ 4 mm as independent variables. When periodontal pockets with depths ≥ 4 mm were observed in at least five sites, the area under the receiver operating characteristic curve, sensitivity, and specificity were 0.73, 0.82, and 0.65, respectively. Similarly, area under the curve of 0.73, sensitivity of 0.41, and specificity of 0.81 were obtained in case the number of periodontal pockets with depths ≥ 4 mm were greater than or equal to 10 (Fig. 4a,b).

Patients with NAFLD who had > 10 periodontal pockets with depths ≥ 4 mm on periodontal examination had significantly increased liver stiffness on both vibration-controlled transient elastography (VCTE) and MRE. Furthermore, in patients with liver stiffness ≥ 3.4 kPa, there were significant differences in salivary and serum *P.* *gingivalis* antibody levels, which may have been related to *P.* *gingivalis*’ endotoxin.

## Discussion

This is a study to demonstrate that the association between NAFLD pathology (especially liver stiffness), *P.* *gingivalis* bacteria contents, and oral findings (PPD). This observational study examining the relationship between periodontal disease and NAFLD pathogenesis produced three major findings. First, it demonstrated that the group with ≥ 0.01% *P.* *gingivalis* in saliva had a higher MRE-determined liver stiffness than the group with < 0.01% *P.* *gingivalis* in saliva. *P.* *gingivalis* migrates relatively easily from the oral cavity into the blood circulation^39,40^; therefore, its endotoxin (present in the oral cavity) can reach the liver^41^.

Second, it found that the group with MRE-determined liver stiffness values of ≥ 3.4 kPa had higher serum *P.* *gingivalis* FDC381 and SU63 antibody levels. *P.* *gingivalis* is an obligate anaerobic gram-negative bacillus with strong adhesion; therefore, it forms a biofilm with other gram-negative bacteria associated with periodontal disease. In addition to producing a proteolytic enzyme called gingipains, *P.* *gingivalis* expresses lipopolysaccharides (which are endotoxins of the outer bacterial membrane) and is an anaerobic bacterium associated with periodontal disease progression. Inflammation caused by the cytokines and proteins produced during *P.* *gingivalis* infection is associated with liver stiffness^42,43^. Furthermore, the histological lesions in NASH are unevenly distributed throughout the liver parenchyma; therefore, liver biopsy errors can result in substantial stratification and staging inaccuracies^44^. We consider it important to perform non-invasive, safe, low-cost, and short-term clinical trials as proof-of-concept studies. Many studies have reported that the steatosis grade could be evaluated using the controlled attenuation parameter^45–50^ (which is based on the properties of ultrasonographic signals acquired with VCTE) or magnetic resonance imaging (MRI) proton density fat fraction^51,52^ (which is an MRI-based method for quantitatively assessing hepatic steatosis and is available for several types of MRI scanners). Moreover, VCTE and MRE have excellent diagnostic abilities for steatosis and fibrosis in patients with NAFLD^53,54^. We consider that non-invasive evaluation of the pathogenesis of NAFLD using VCTE and MRI may replace invasive methods, such as liver biopsy. For this reason, we included non-invasive methods for assessing NASH/NAFLD progression as secondary endpoints and compared the results with those derived from liver biopsies.

Third, the group with ≥ 10 periodontal pockets with depths ≥ 4 mm had increased liver stiffness compared to the group with < 10 periodontal pockets with depths ≥ 4 mm. The presence of more than 10 periodontal pockets with depths > 4 mm suggests high morbidity in patients with periodontal disease^55^. The factor involved in PPD and liver stiffness in this study may have been age, and not sex. With increasing age, the prevalence of periodontal disease rises, and periodontal pockets become deeper^56^. However, the average age of the patients was 53.0 years, and age was not associated with the other examined factors in this study.

Inflammation caused by the cytokines and proteins produced in response to *P.* *gingivalis* infection is associated with liver stiffness^42,43^. Therefore, the amount of bacteria in the oral cavity and serum antibody titers were examined in this cross-sectional study, and the relationship between periodontal disease and liver stiffness in patients with NAFLD was evaluated.

NAFLD is common in patients with metabolic abnormalities, such as type 2 diabetes and obesity; its prevalence is rapidly increasing globally with the growing obese population^5^. The main risk associated with this disease is that it progresses from liver cirrhosis to liver cancer over a long time without manifestation of accompanying subjective symptoms, and mortality due to cerebral or myocardial infarction is also high. It is thought that the pathology in patients with NASH progresses as periodontopathic bacteria enter the blood, leading to inflammation in the fatty liver. Therefore, it is necessary to actively interfere with the link between periodontal disease and NAFLD.

The strength of this study lies in the use of non-invasive MRI (in place of liver biopsy) to assess changes in liver fat content. Importantly, this non-invasive method revealed a seemingly mutual relationship between *P.* *gingivalis* and liver stiffness.

The study has a certain limitation. It would be important to differentiate NASH from NAFLD in the patients enrolled in this study; however, currently, biopsy is not used to diagnose and differentiate liver tissue.

In summary, patients with NAFLD who had *P.* *gingivalis* infection and deeper periodontal pockets had higher liver stiffness values than those who did not have periodontal disease.

Furthermore, the serum antibody titers for *P.* *gingivalis* FDC381 and SU63 were high in the patient group with increased liver stiffness, and logistic regression analysis suggested that periodontal disease is associated with liver stiffness. These results indicate that periodontal disease may be associated with NAFLD. As this was a cross-sectional study, a causal relationship could not be ascertained. Additional randomized controlled trials are needed to investigate the relationship between periodontal disease and NAFLD, and further investigation of patients with NAFLD who do not have periodontal disease is needed. As this study showed that liver fibrosis and periodontal disease are likely to be related, future interventional studies with treatment for fibrosis as an endpoint will be necessary.

## Methods

The recruitment process for this study was carried out at Kanagawa Dental University Yokohama Clinic, Kanagawa Dental University, Iwasaki Internal Medicine Clinic, and Yokohama City University Hospital Cohort. Patient recruitment process was conducted over a period of 8 h per day, 5 days a week. Eligible patients were screened by the attending physicians and deputy researchers (gastroenterologists and periodontists). The design of our research required patient involvement; specifically, the development of the research question was based on experiences of the patients. The Kanagawa Dental University Committee for Research Screening approved this study (approval number: 323; approval date: August 21, 2015). All procedures were performed in accordance with the tenets of the Declaration of Helsinki.

Each participant was examined to assess the amount of periodontal disease-causing bacteria (e.g., *P.* *gingivalis,* *T.* *fosythensis,* *and* *T.* *denticola*) present in oral cavity, the degree of infection, and periodontal disease severity. All examinations were performed by dentists affiliated with Kanagawa Dental University Hospital and Kanagawa Dental University Yokohama Clinic. The amount of *P.* *gingivalis* in saliva was measured using quantitative PCR, and the infection level was evaluated using serum immunoglobin G (IgG) antibody titer tests for *P.* *gingivalis* FDC381 and SU63 based on enzyme-linked immunosorbent assays. The severity of periodontal disease was determined by assessing the PPD, clinical attachment level, level of gingival BOP at six sites per tooth using a calibrated periodontal probe (HU-FRIEDY PQ-OW Thin Williams, Hu-Friedy , Chicago, IL, USA), and the stability of the teeth. The patients with NAFLD recruited in this study met the inclusion and exclusion criteria listed in Supplementary Tables S1 and S2, respectively. Fatty liver, steatosis grade, and fibrosis stage were evaluated using non-invasive methods such as ultrasonography (VCTE) and MRI.

### Registration

For patients judged to be eligible, the principal investigator or collaborator filled out a patient registration form with the necessary information and sent these data to the Patient Enrollment Center (Yokohama City University). The Patient Enrollment Center confirmed the patient's eligibility based on the registration form, registered the patient, and informed the main researcher or collaborator of the patient’s identification and assignment numbers. All participants provided informed consent to participate in the study prior to the commencement of the study.

### Sample size calculation

A retrospective analysis of patients with NAFLD and periodontitis (*P.* *gingivalis* ratio < 0.01% vs. *P.* *gingivalis* ratio ≥ 0.01%) in Yokohama City University Hospital showed a mean liver stiffness of 2.8 and 3.6 in the *P.* *gingivalis* < 0.01% and *P.* *gingivalis* ≥ 0.01% groups, respectively, on MRE. We decided to calculate the number of patients required for a proper analysis of the variance t-test based on these data. Considering the liver stiffness (determined with MRE) in the *P.* *gingivalis* < 0.01% and *P.* *gingivalis* ≥ 0.01% groups to be 2.8 and 3.6, respectively, with a common SD of 1.8, a total of 159 patients were needed to reach an 80% statistical power with a two-sided significance level of 5%. To compensate for dropouts, we proposed an increase in the number of patients to 164.

### Statistical analyses

In this study, statistical analyses were performed mainly for the following items. As primary analysis, stratification analysis was performed using the *P.* *gingivalis* bacterial content, MRE, and periodontal pockets as indexes. At the secondary endpoint, the *P.* *gingivalis* IgG antibody titer in the blood was required for each participant, and the paired Wilcoxon signed-rank test was performed. Data are presented as means ± SDs or numbers (percentages). The significance level was set at p < 0.05. We used statistical software for all analyses (SAS version 9.4, SAS Institute, Cary, NC, USA).

### Trial steering and data monitoring committees

The pilot committee consisted of three people appointed by independent clinical and basic researchers (general and palliative care specialists and statisticians at Yokohama City University School of Medicine). They provided overall supervision and ensured all registered examinations. These investigators were anonymous and randomly selected. The Independent Data Monitoring Committee comprised two members of staff from the Department of Biostatistics, Yokohama City University School of Medicine. The management team monitored the monthly progress of patients and data with phone, email, and/or web conferencing. If the oversight committee determined that field monitoring was necessary, a member of the Independent Data Monitoring Committee visited the site for face-to-face monitoring.

### Ethics declarations

This study involves human participants and was approved by the Kanagawa Dental University Committee for Research Screening (Approval No: 323). All procedures were performed in accordance with the Declaration of Helsinki.

### Consent to participate/Consent to publish

All participants provided informed consent to participate in the study prior to initiation of the study. Participants also signed a form indicating their agreement to the presentation of their data at conferences and in papers in a manner that does not disclose their personal information.

## Data availability 

All data generated or analyzed during this study are included in this published article and its Supplementary Information files.

## Supplementary Information

Supplementary Tables.
