# Supplementary material for: A cross-sectional study assessing the relationship between non-alcoholic fatty liver disease and periodontal disease
Source: Sci Rep. 2022 Aug 10;12:13621. doi: 10.1038/s41598-022-17917-2 (PMC9365789; doi:10.1038/s41598-022-17917-2)
Supplement: Supplementary file 1 — Supplementary Tables. [file 41598_2022_17917_MOESM1_ESM.docx]

**Supplemental Material**

**Table S1** PERION inclusion criteria

| Criterion | Description of the inclusion criteria |
| --- | --- |
| Sex and age | Males and females; 20–85 years of age |
| Diet and exercise therapy | Patients with NAFLD who did not respond to a 3-month diet and exercise therapy |
| ALT levels | Patients with an ALT level of >40 IU/L at the start of this study |
| Fatty liver | Patients with a diagnosis of fatty liver based on abdominal ultrasonography^a^ |
| Steatosis grade | Patients with the equivalent of steatosis grade ≥1 on CAP (using FibroScan) and/or PDFF (using MRI)^b^ |
| Fibrosis stage | Patients with the equivalent of fibrosis stage <4 on VCTE (FibroScan) and/or MRE^c^ |
| Alcohol consumption | Patients with no habitual alcohol consumption (i.e., consumption of ethanol >30 g/day in men and >20 g/day in women) |
| Periodontitis | Patients with chronic moderate periodontitis (periodontal pocket depth of >4 mm at >10 sites） |
| Other | Patients who can provide written consent to participate in this research in person, follow instructions during participation in this research, undergo protocol-specified physical and other examinations, and report symptoms or events |

ALT, alanine aminotransferase; CAP, controlled attenuation parameter; MRE, magnetic resonance elastography; MRI, magnetic resonance imaging; NAFLD, nonalcoholic fatty liver disease; PERION, periodontal treatment for NAFLD; PDFF, proton density fat fraction; VCTE, vibration-controlled transient elastography.

^a^ Criteria of fatty liver, as defined by the existence of hepatorenal echo contrast.

^b^ Defined by CAP ≥236 dB/m or PDFF ≥5.2%.

^c^ Defined by VCTE <14 kPa or MRE <6.7 kPa

**Table S2** PERION exclusion criteria

| Criterion | Description of the exclusion criteria |
| --- | --- |
| Liver comorbidity | Patients with any other concurrent liver disease, such as hepatitis C, hepatitis B, or autoimmune hepatitis |
|  | Patients with drug-induced symptomatic NAFLD |
| Other comorbidities | Patients with a concurrent or past history of any serious cardiac, vascular, hematological, respiratory, hepatic, renal, gastrointestinal, or neuropsychiatric disease |
|  | Patients with a history of abdominal or gastrointestinal surgery, except appendicitis |
| Medication | Patients with any change to their oral medications within 3 months before informed consent |
|  | Patients with diabetes mellitus being treated with insulin injections |
| Other | Patients who participated in any other clinical study and received study treatment within 1 month before the start of this research (counted from the first day of study medication) |
|  | Breastfeeding women or women with possible pregnancy |
|  | Other patients who are inappropriate as participants in this research in the opinion of the principal investigator, etc. |

NAFLD, nonalcoholic fatty liver disease; PERION, periodontal treatment for NAFLD

**Table S3** Genus levels in the oral microbiota of NAFLD patients with periodontitis

|  | Number of PPDs ≥4 mm | |  |
| --- | --- | --- | --- |
|  | <5 | ≥5 |  |
| *Genus* | (n=85) | (n=79) | p-value |
| *Filifactor* | 0.1（0.2） | 0.2（0.2） | 0.0003 |
| *Treponema* | 0.2（0.2） | 0.7 (1.3) | 0.0003 |
| *Mannheimia* | 2.3 (1.7) | 1.5 (1.4) | 0.0006 |
| *Tannerella* | 0.1 (0.2) | 0.2 (0.2) | 0.002 |
| *Actinomyces* | 2.3 (1.6) | 1.8 (1.1) | 0.009 |
| *Haemophilus* | 4.4 (3.5) | 3.3 (2.) | 0.02 |
| *Cohnella* | 0.9 (0.9) | 0.6 (0.5) | 0.02 |
| *Aggregatibacter* | 0.2 (0.3) | 0.3 (0.3) | 0.03 |
| *Porphyromonas* | 3.3 (2.8) | 4.6 (4.1) | 0.03 |
| *Actinobacillus* | 0.5 (0.6) | 0.3 (0.4) | 0.03 |
| *Bacteroides* | 1.6 (1.2) | 1.2 (1.0) | 0.04 |
| *Selenomonas* | 0.7 (0.6) | 0.9 (0.9) | 0.05 |

PPD, periodontal probing depth.

**Table S4** Diversity and abundance of species in the liver of NAFLD patients with periodontitis

|  |  | Number of PPDs ≥4 mm | | |  |  |
| --- | --- | --- | --- | --- | --- | --- |
|  |  | <5 |  | ≥5 |  |  |
|  |  | (n=81) |  | (n=76) |  | p-value |
| Shannon index |  | 2.8 (0.2) |  | 2.8 (0.2) |  | 0.4 |
| Chao1 |  | 215 (88) |  | 233 (110) |  | 0.2 |

PPD, periodontal probing depth.

**Table S5** Factors associated with NAFLD in advanced fibrosis

| **Covariate** | **Odds ratio** | **95% Confidence level** | **P value** |
| --- | --- | --- | --- |
| Endotoxin | 0.924 | 0.835-1.023 | 0.105 |
| Weight (kg) | 1.058 | 1.012-1.107 | 0.006 |
| Platelet count (×10^4/μL) | 1.023 | 0.951-1.100 | 0.489 |
| AST (U/L) | 0.982 | 0.952-1.012 | 0.22 |
| type VII collagen 7s (ng/mL) | 0.605 | 0.309-1.181 | 0.13 |
| BOP | 0.956 | 0.925-0.988 | 0.002 |
| *P. gingivalis* in saliva (cell/ml) | 1 | 1.000-1.000 | 0.483 |
| P.g FDC381 | 0.834 | 0.616-1.129 | 0.205 |
| P.g SU63 | 1.016 | 0.677-1.523 | 0.941 |
| IMT mean R | 0.117 | 0.003-4.623 | 0.253 |
| IMT mean L | 0.113 | 0.003-4.270 | 0.232 |
| PPD (mm) | 1.184 | 1.006-1.393 | 0.025 |

We also constructed receiver operating characteristic curves for patients with at least 0.01% of *P. gingivalis* in saliva and the number of sites with PPDs of ≥4 mm as independent variables. When PPDs of ≥4 mm were observed in at least 5 sites, the area under the receiver operating characteristic curve was 0.73, sensitivity was 0.82, and specificity was 0.65. Similarly, an area under the curve of 0.73, sensitivity of 0.41, and specificity of 0.81 were obtained when the number of PPDs of ≥4 mm was 10 or higher (Figure 4a, b).
